# Incidence, risk factors, and outcomes of second neoplasms in patients with acute promyelocytic leukemia: the PETHEMA-PALG experience

**DOI:** 10.1007/s00277-023-05582-y

**Published:** 2023-12-19

**Authors:** Marta Sobas, Wanda Knopinska-Posluszny, Beata Piątkowska-Jakubas, Flor García-Álvarez, María Elena Amutio Díez, Mar Caballero, David Martínez-Cuadrón, Eliana Aguiar, Jose González-Campos, Ana Garrido, Lorenzo Algarra, Olga Salamero, Javier de la Serna, Maria Jose Sayas, Manuel Mateo Perez-Encinas, Susana Vives, Belén Vidriales, Jorge Labrador, Ana Inés Prado, Lucía Celebrin, Jiri Mayer, Joana Brioso, Almudena de Laiglesia, Juan Miguel Bergua, Maria Luz Amigo, Carlos Rodriguez-Medina, Marta Polo, Agnieszka Pluta, Edyta Cichocka, Marek Skarupski, Miguel A Sanz, Agnieszka Wierzbowska, Pau Montesinos

**Affiliations:** 1 Department of Hematology, Blood Neoplasm and Bone Marrow Transplantation, Wroclaw Medical University, Wroclaw, Poland; 2 Department of Hematology and Transplantology, Gdynia, Poland; 3 Collegium Medicum Jagiellonian University, Krakow, Poland; 4 Hospital Central de Asturias, Oviedo, Spain; 5 Hospital de Cruces, Barakaldo, Spain; 6 Hospital Insular de Las Palmas, Las Palmas de Gran Canaria, Spain; 7 Hospital Universitario i Politècnico la Fe, Valencia, Spain; 8 Centro Hospitalar Săo Joăo, Porto, Portugal; 9 Hospital U. Virgen del Rocio, Sevilla, Spain; 10 Hospital Sant Pau, Barcelona, Spain; 11 Hospital General de Albacete, Albacete, Spain; 12 Hospital U. Vall D’Hebron, Barcelona, Spain; 13 Hospital 12 de Octubre, Madrid, Spain; 14 Hospital Dr. Peset, Valencia, Spain; 15 University Hospital, Santiago de Compostela, Spain; 16 Hospital U. Germans Trias i Pujol ICO, Badalona, Spain; 17 University Hospital of Salamanca (CAUSA/IBSAL) and Center for Biomedical Research in Network of Cancer (CIBERONC), Salamanca, Spain; 18 Department of Hematology, Research Unit, Hospital Universitario de Burgos, Facultad de Ciencias de la Salud, Universidad Isabel I, Burgos, Spain; 19 Hospital Maciel, Montevideo, Uruguay; 20 Hospital Tornú, Buenos Aires, Argentina; 21 University Hospital Brno, Masaryk University, Brno, Czechia; 22 Hospital de Santa Maria, Lisboa, Portugal; 23 Hospital Puerta de Hierro, Madrid, Spain; 24 Hospital San Pedro de Alcántara, Caceres, Spain; 25 Hospital Morales Meseguer, Murcia, Spain; 26 Hospital Universitario de Gran Canaria Doctor Negrin, Las Palmas de Gran Canaria, Spain; 27 Hospital Clínico San Carlos, Madrid, Spain; 28 Medical University of Lodz, Lodz, Poland; 29 Rydygiera Hospital, Torun, Poland; 30 Faculty of Pure and Applied Mathematics, Wrocław University of Science and Technology, Wroclaw, Poland; 31 Department of Mathematics and Computer Science, Eindhoven University of Technology, 5612 AZ Eindhoven, The Netherlands

**Keywords:** Acute promyelocytic leukemia, Second neoplasms, Chemotherapy based and chemotherapy free regimens, Risk factors, Outcomes

## Abstract

The most important challenges in acute promyelocytic leukemia (APL) is preventing early death and reducing long-term events, such as second neoplasms (s-NPLs). We performed a retrospective analysis of 2670 unselected APL patients, treated with PETHEMA “chemotherapy based” and “chemotherapy free” protocols. Only de novo APL patients who achieved complete remission (CR) and completed the three consolidation cycles were enrolled into the analysis. Out of 2670 APL patients, there were 118 (4.4%) who developed s-NPLs with the median latency period (between first CR and diagnosis of s-NPL) of 48.0 months (range 2.8–231.1): 43.3 (range: 2.8–113.9) for s-MDS/AML and 61.7 (range: 7.1–231.1) for solid tumour. The 5-year CI of all s-NPLs was of 4.43% and 10 years of 7.92%. Among s-NPLs, there were 58 cases of s-MDS/AML, 3 cases of other hematological neoplasms, 57 solid tumours and 1 non-identified neoplasm. The most frequent solid tumour was colorectal, lung and breast cancer. Overall, the 2-year OS from diagnosis of s-NPLs was 40.6%, with a median OS of 11.1 months. Multivariate analysis identified age of 35 years (hazard ratio = 0.2584; *p* < 0.0001) as an independent prognostic factor for s-NPLs. There were no significant differences in CI of s-NPLs at 5 years between chemotherapy-based vs chemotherapy-free regimens (hazard ratio = 1.09; *p* = 0.932). Larger series with longer follow-up are required to confirm the potential impact of ATO+ATRA regimens to reduce the incidence of s-NPLs after front-line therapy for APL.

**Supplementary Information:**

The online version contains supplementary material available at 10.1007/s00277-023-05582-y.

## Introduction

Acute promyelocytic leukaemia (APL) is the most curable subtype of adult acute myeloid leukaemia (AML) with a complete remission (CR) rate greater than 90% [1–12]. This has been achievable due to improvements in the diagnostic tools and supportive care, as well as the introduction of all-*trans*-retinoic acid (ATRA) [1–9] and arsenic trioxide (ATO) into the treatment [10–12]. Nowadays, a part of solving early death, another important challenge in the treatment of APL patients is to reduce the long-term events, such as second neoplasms (s-NPLs).

Though the reported incidence of s-NPLs after APL therapy is relatively low, their outcome seems very poor [13–23]. However, there are only a few studies analysing the risk factors for the development of s-NPLs after APL therapy. Among chemotherapy-based regimens, topoisomerase II inhibitors and alkylating drugs are proved to be associated with s-MDS/AML development [24, 25], but these drugs are not used in APL. It is supposed that chemotherapy-free regimens, based on ATRA plus ATO, could reduce the number of s-MDS/AML in APL survivals [20–22]. On the other hand, there is a close correlation between long-term environmental arsenic exposure and an increase in solid tumours incidence [26, 27]. Of note, there is still scarce information in the literature concerning s-NPLs after chemotherapy-free APL regimens [20–22].

There are two main s-NPLs categories in APL patients: solid tumours and second myelodysplastic syndrome (s-MDS) or acute myeloid leukemia (s-AML). Previous multicentre study by the PETHEMA (Programa para el Tratamiento de Hemopatías Malignas) group characterized patients with s-MDS/AML developed after chemotherapy-based (AIDA) APL protocols. There are still few data concerning the incidence and outcomes of solid tumours after APL therapy [19–23].

This retrospective multicentre study aims to analyse the incidence, risk factors and clinical outcomes of s-NPLs in de novo APL patients treated with 5 consecutive PETHEMA protocols.

## Methods

**Fig. 1:**
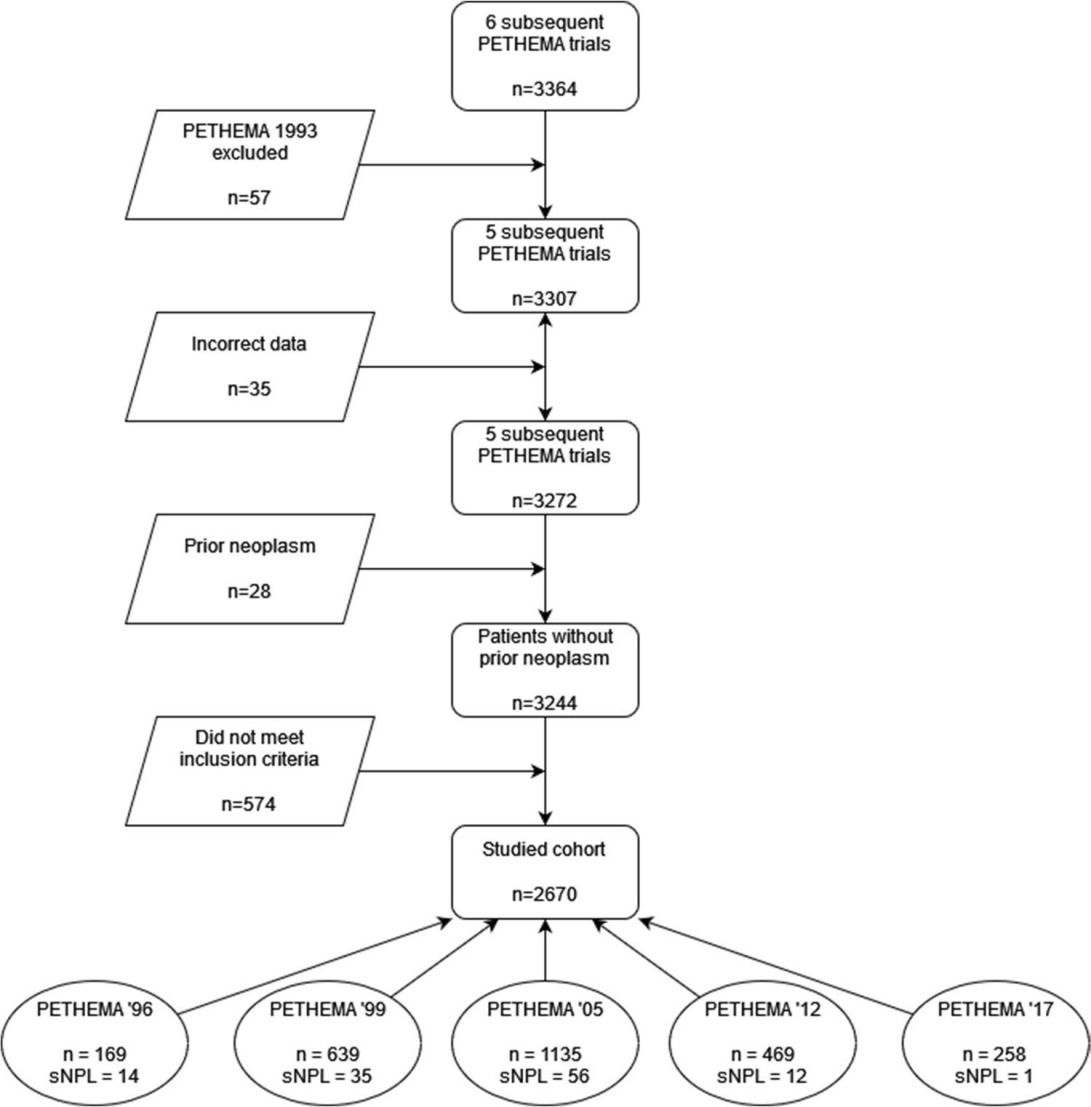
**Patient’s selection for the study.** Diagnosis of APL was confirmed by the presence of t(15;17) in conventional cytogenetic analysis, and/or detection of the PML/RARa with fluorescence in situ hybridization (FISH) or standardized reverse transcription-polymerase chain reaction (RT-PCR) analysis with a sensitivity of 103-104. Only *de novo* APL patients who achieved complete remission (CR) and who completed the three consolidation cycles were enrolled into the analysis. Patients with APL second to prior neoplasm, as they received chemo and/or radiotherapy before diagnosis of APL, were excluded from the analysis. Patients were treated in Spain, the Netherlands, Argentina, Uruguay, Belgium, Czech Republic, Portugal (PETHEMA registry) and Poland (PALG, Polish Adult Leukemia Group registry); n: number, sNPL: second neoplasm

We performed a retrospective analysis on 2670 unselected newly diagnosed APL patients, who were treated according to PETHEMA “chemotherapy based” (APL1996, 1999, 2005, 2012 and 2017 for high risk) and “chemotherapy free” regimen (2017 for low/intermediate risk) between 11.1996 and 11.2021. Treatment protocols are summarized in Table 1 [4, 5, 28, 29] and study selection in Fig. 1. The study was conducted in accordance with the Declaration of Helsinki. Informed written consent to follow the treatment according to PETHEMA protocol was obtained.

**Table 1:** Therapy of APL in the consecutive PETHEMA trials

Protocol	Risk group		Consolidation regimen				
			Consolidation 1	Consolidation 2	Consolidation 3		
**1999**	Low	**INDUCTION REGIMEN**: AIDA	Ida 5 mg/m^2^ × 4d	MTZ 10 mg/m^2^ × 5d	Ida 12 mg/m^2^ × 1d		**MAINENACE THERAPY (2 years)**: ATRA + MP + MTX
	Intermediate/High		Ida 7 mg/m^2^ × 4d ATRA 45 mg/m^2^ × 15d	MTZ 10 mg/m^2^ × 5d ATRA 45 mg/m^2^ × 15d	Ida 12 mg/m^2^ × 2d ATRA 45 mg/m^2^ × 15d		
**2005**	Low		Ida 5 mg/m^2^ × 4d ATRA 45 mg/m^2^ × 15d	MTZ 10 mg/m^2^ × 3d ATRA 45 mg/m^2^ × 15d	Ida 12 mg/m^2^ × 1d ATRA 45 mg/m^2^ × 15d		
	Intermediate		Ida 7 mg/m^2^ × 4d ATRA 45 mg/m^2^ × 15d	MTZ 10 mg/m^2^ × 3d ATRA 45 mg/m^2^ × 15d	Ida 12 mg/m^2^ × 2d ATRA 45 mg/m^2^ × 15d		
	High		Ida 5 mg/m^2^ × 4d ATRA 45 mg/m^2^ × 15d ARA-C 1 g/m^2^ × 4d	MTZ 10 mg/m^2^ × 5d ATRA 45 mg/m^2^ × 15d	Ida 7 mg/m^2^ × 1d ATRA 45 mg/m^2^ × 15d ARA-C 150 mg/m^2^ ×5d		
**2012**	Low		Ida 5 mg/m^2^ × 4d ATRA 45 mg/m^2^ × 15d	MTZ 10 mg/m^2^ × 3d ATRA 45 mg/m^2^ × 15d	Ida 12 mg/m^2^ × 1d ATRA 45 mg/m^2^ × 15d		
	Intermediate		Ida 5 mg/m^2^ × 4d ATRA 45 mg/m^2^ × 15d ARA-C 500 mg/m^2^/d × 4d	MTZ 10 mg/m^2^ × 3d ATRA 45 mg/m^2^ × 15d	Ida 12 mg/m^2^ × 2d ATRA 45 mg/m^2^ × 15d		
	High		Ida 5 mg/m^2^ × 4d ATRA 45 mg/m^2^ × 15d ARA-C 1 g/m^2^ × 4d	MTZ 10 mg/m^2^ × 5d ATRA 45 mg/m^2^ × 15d	Ida 12 mg/m^2^ × 1d ATRA 45 mg/m^2^ × 15d ARA-C 500 mg/m^2^ ×4d		
**2017**	Low	**INDUCTION REGIMEN:** ATRA+ATO	ATRA 45 mg/m^2^ × 14d (week 1–2 and 5–6) ATO 0,15 mg/kg week 1–4 (5d*)	ATRA 45 mg/m^2^ × 14d (week 9–10 and 13–14) ATO 0,15 mg/kg week 8–12 (5d*)	ATRA 45 mg/m^2^ × 14d (week 17–18 and 21–22) ATO 0,15 mg/kg week 17–20 (5d*)	ATRA 45 mg/m^2^ × 14d (week 25–26) ATO 0,15 mg/kg week 25-28 (5d*)	**STOP**
	Intermediate						
	High (>=60 and < 70 years old)	**INDUCTION REGIMEN**: AIDA	Ida 5 mg/m^2^ × 4d ATRA 45 mg/m^2^ × 15d	MTZ 10 mg/m^2^ × 3d ATRA 45 mg/m^2^ × 15d	Ida 12 mg/m^2^ × 1d ATRA 45 mg/m^2^ × 15d		**MAINENACE THERAPY (2 years)**: ATRA + ATO
	High (<60 years old)		Ida 5 mg/m^2^ × 4d ATRA 45 mg/m^2^ × 15d ARA-C 1 g/m^2^ × 4d	MTZ 10 mg/m^2^ × 5d ATRA 45 mg/m^2^ × 15d	Ida 12 mg/m^2^ × 1d ATRA 45 mg/m^2^ × 15d ARA-C 500 mg/m^2^ ×4d		

*ATRA*, all-trans retinoic acid; *IDA*, idarubicin; *MTZ*, mitoxantrone, Ara-C-cytarabine; *MP*, 6-mercaptopurine; *MTX*, methotrexate 5d* for example from Monday to Friday

Definitions of CR, molecular remission and persistence, hematological and molecular relapse have been reported previously [30, 31]. Diagnosis of s-MDS/AML was made according to the WHO criteria [32]. APL relapse was ruled out by cytomorphological and cytogenetic/molecular assessments [30, 31]. The diagnosis of other hematological malignancies and solid tumours was confirmed by histopathological analysis of the excised tissues.

The last patient follow-up was updated on December 2022. The following data was collected at diagnosis: age, gender, ECOG (Eastern Cooperative Oncology Group) performance status score, platelet and leucocyte (WBC) counts, Sanz risk score, fever and coagulopathy, FLT3 (FMS-like tyrosine kinase 3) — internal tandem duplication (ITD), karyotype: t(15;17) vs complex karyotype. Complex karyotype was defined as karyotype with minimum 3 independent abnormalities [33]. Survival and cumulative incidence curves were determined using non-parametric methods using Kaplan-Meier estimators [34]. Differences between curves are done by log-rank test and *p*-value is given [35]. The probability of developing s-NPL was also estimated by cumulative incidence and the estimated variance was calculated using Aalen–Johansen estimators [36, 37]. The cumulative incidence of n-NPL was calculated from the date of CR. For the cumulative incidence analysis, death in CR and relapse of APL were considered competing causes of failure. Multivariate analysis was performed using the Cox proportional hazards model [38]. Overall survival (OS) was calculated from the date of development of s-NPL. The chi-2 test, with Yates’ correction if necessary, and Mann-Whitney *U* test and *t* test were used to analyse differences in the distribution between patient subsets of categorical and continuous nonparametric and parametric variables, respectively. Median lifetimes were determined taking into account censored data. Even if median survival has been reached in a group, it was not possible to calculate complete confidence intervals for those median values. The odds ratio was calculated by median-unbiased estimation. Confidence intervals are calculated using exact methods (mid-p). Characteristics selected for inclusion in the multivariate analysis were those for which there was a significant association in univariate analysis (*p* .05). All *p* values reported are two-sided. All tests were performed in R version 4.2.1.

## Results

### Patient selection

Between November 1996 and November 2021, 3364 APL patients were registered and were intended to be treated according to PETHEMA protocols in Spain, the Netherlands, Argentina, Uruguay, Belgium, Czech Republic, Portugal and Poland. Information on enrolment, eligible patients, lost to follow-up, and exclusion from analysis is shown in a flow diagram (Fig. 1). Of them, there were 2670 *de novo* APL patients who achieved complete remission and completed induction and all consolidation cycles. Patients included into the study, were treated according to subsequent PETHEMA protocols: LPA1996 (*n*=169), LPA1999 (*n*=639), LPA2005 (*n*=1135), LPA2012 (*n*=469), LPA2017 (*n*=258). Main clinical and analytical characteristics of patients included into analysis are presented in a Table 2.

**Table 2:** Main characteristics of analyzed APL patients according to development of different type of s-NPLs

Features	No s-NPL	s-MDS/AML	Other hematological neoplasms	Solid tumour
**No. of patients**	2552	58	3	57
**PETHEMA trial**				
LPA1996	155	5	1	8
LPA1999	604	16	1	18
LPA2005	1080	29	1	25
LPA2012	456	8	0	5
LPA2017	257	0	0	1
**Age, years**				
⩽ 35	835	9	0	7
>35	1713	48	3	49
ND	4	1	0	1
**Sex**				
Male	1247	23	3	24
Female	1305	35	0	33
**WBC count, × 10**^**9**^ **/L**				
⩽ 10	1938	44	1	49
>10	606	14	2	8
NA	8	0	0	0
**Platelet count, × 10**^**9**^ **/L**				
⩽ 40	1748	41	3	37
>40	78717	170	00	191
**NA**				
**Sanz risc score**				
Low	610	13	0	18
Intermediate	1336	29	1	31
High	5988	142	20	80
**NA**				
**Karyotype**				
No data	754	14	0	13
t(15;17)	1215	31	1	32
t(15;17)+others	368	8	2	10
Normal	215	5	0	2
**FAB subtype**				
Hypergranular	1980	43	2	47
Microgranular	354	9	1	9
No data	218	6	0	1
**PML/RARA isoform**				
BCR-1	937	19	2	18
BCR2	57	1	0	2
BCR3	727	17	1	25
BCR1/BCR2	136488	411	0	15
ND				
**FLT3-ITD**				
Negative	543	9	0	12
Positive	219	5	0	3
Not done	1790	44	3	42

### Incidence of second NPLs

Median follow-up of the series was 77 months (range, 17 to 158 months) from diagnosis. Out of 2670 APL patients, 118 (4.4%) developed s-NPLs during follow-up with the median latency period (between first CR and diagnosis of s-NPL) of 48.0 months (range: 2.8–231.1): 43.3 (range: 2.8–113.9) for s-MDS/AML and 61.7 (range: 7.1–231.1) for solid tumour (excluding other hematological neoplasms).

The 5-year CI of all s-NPLs was of 4.43% and 10 years of 7.92%. The CI according to protocols (APL1996, 1999, 2005, 2012, 2017) was as follow: 4.33% vs 3.69% vs 4.91% vs 5.14% vs 1.27% (5 years) and 8.94% vs 6.07% vs 9.61% vs 8.83% vs incomplete observation (10 years) (Table 3, Supplementary Fig. 1).

**Table 3:** Comparison of cumulative incidence rates of s-NPLs according to type of second neoplasm, PETHEMA protocol, patient’s characteristic

Characteristics		No. of s-NPLs (%)	5-year CI	10-year CI	*P*-value	HR	95% CI
All patients		118 (4.4%)	4.43%	7.92%	-	-	-
Type of s-NPLs	s-MDS/AML	58 (49%)	2.81%	4.03%	-	-	
	Other hematological	3 (2.5%)	0.12%	0.21%	-	-	
	Unclassified	1 (0.9%)	-	-	-	-	
	s-solid	56 (47.5%)	1.55%	3.71%	-	-	
PETHEMA protocol	APL1996 (*n*=169)	14 (8.3%)	4.33%	8.94%	0.448	-	-
	APL1999 (*n*=639)	35 (5.5%)	3.69%	6.07%			
	APL2005 (*n*=1135)	56 (4.9%)	4.91%	9.61%			
	APL2012 (*n*=469)	12 (2.9%)	5.14%	8.83%			
	APL2017 (*n*=258)	1 (0.4%)	1.27%	NA			
Age, years	>35	100	1.65%	2.36%	< 0.0001	0.2584	(0.1477; 0.4518 )
	<=35	16	5.67%	10.74%			
Sex	M	50	3.46%	7.30%	0.183	-	-
	F	68	5.35%	8.52%			

Among s-NPLs, there were 58 cases of s-MDS/AML, 3 cases of other hematological neoplasms (2 myeloproliferative neoplasms and 1 Hodgkin lymphoma), 57 solid tumours and 1 non-identified neoplasm. CI according to the type of s-NPLs is presented in Table 3, Supplementary Fig. 2.

**Fig. 2:**
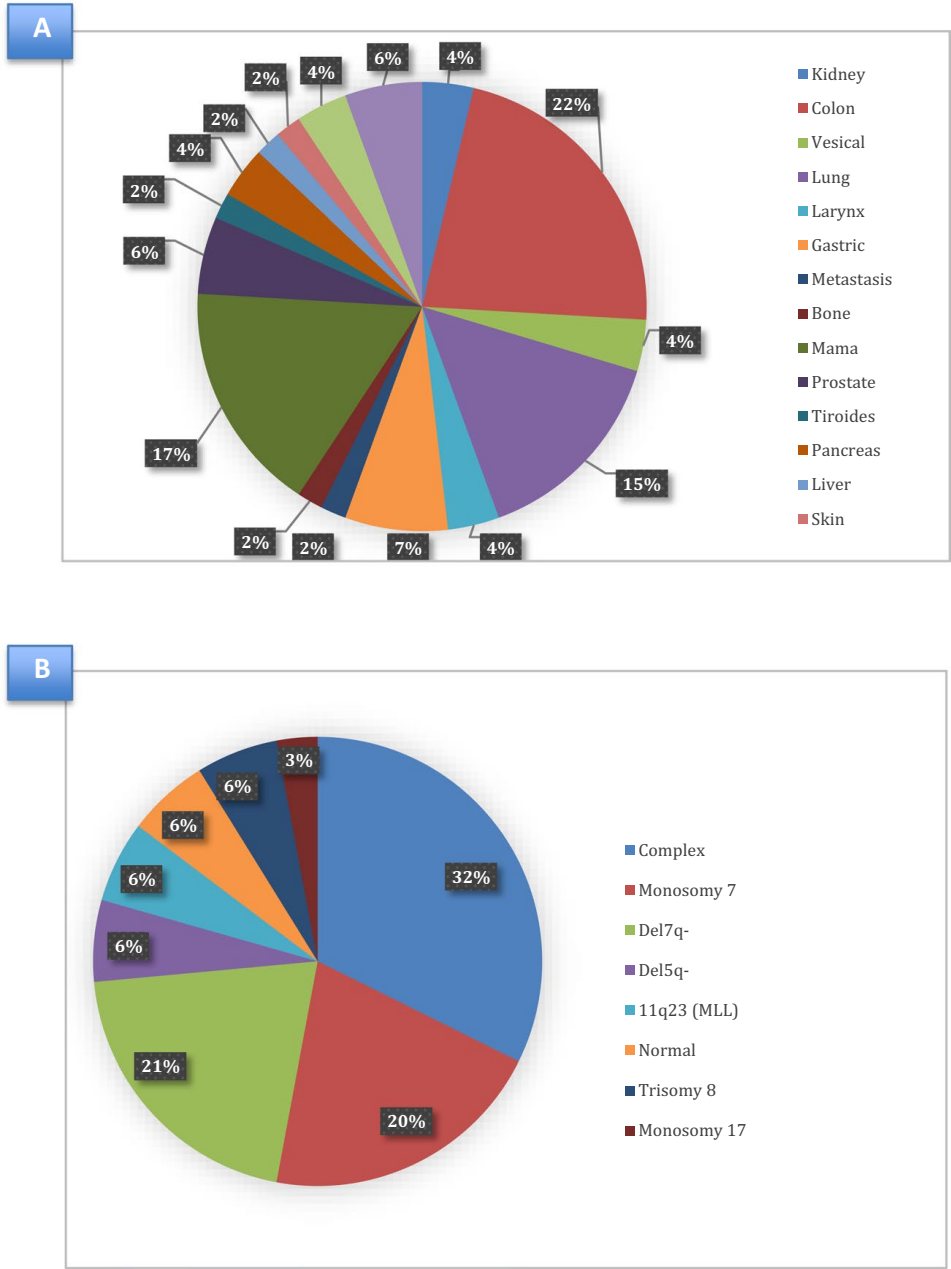
**Characteristics of second neoplasms.** (A) Second solid tumour types. (B) Cytogenetical abnormalities in second MDS/AML

Information concerning the type of solid tumour was available in 54/57 (94.7%) patients. The most frequent solid tumour was colorectal, followed by lung and breast cancer (Fig. 2A).

Regarding s-MDS/AML, there was information concerning karyotype abnormalities in 34/58 cases (58.6%) and the most frequent cytogenetic abnormalities were complex karyotype, monosomy of 7, and del 7q (Fig. 2B).

### Risk factors for development of s-NPLs

In patients developing s-NPLs there was a slight predominance of female sex (57.6%) and the median age at time of diagnosis of APL was higher vs APL without s-APLs: 55 (range from 13 to 84) vs 43 (range from 2 to 87) years. There were no differences in the Sanz risk score distribution (Table 2).

**Fig. 3:**
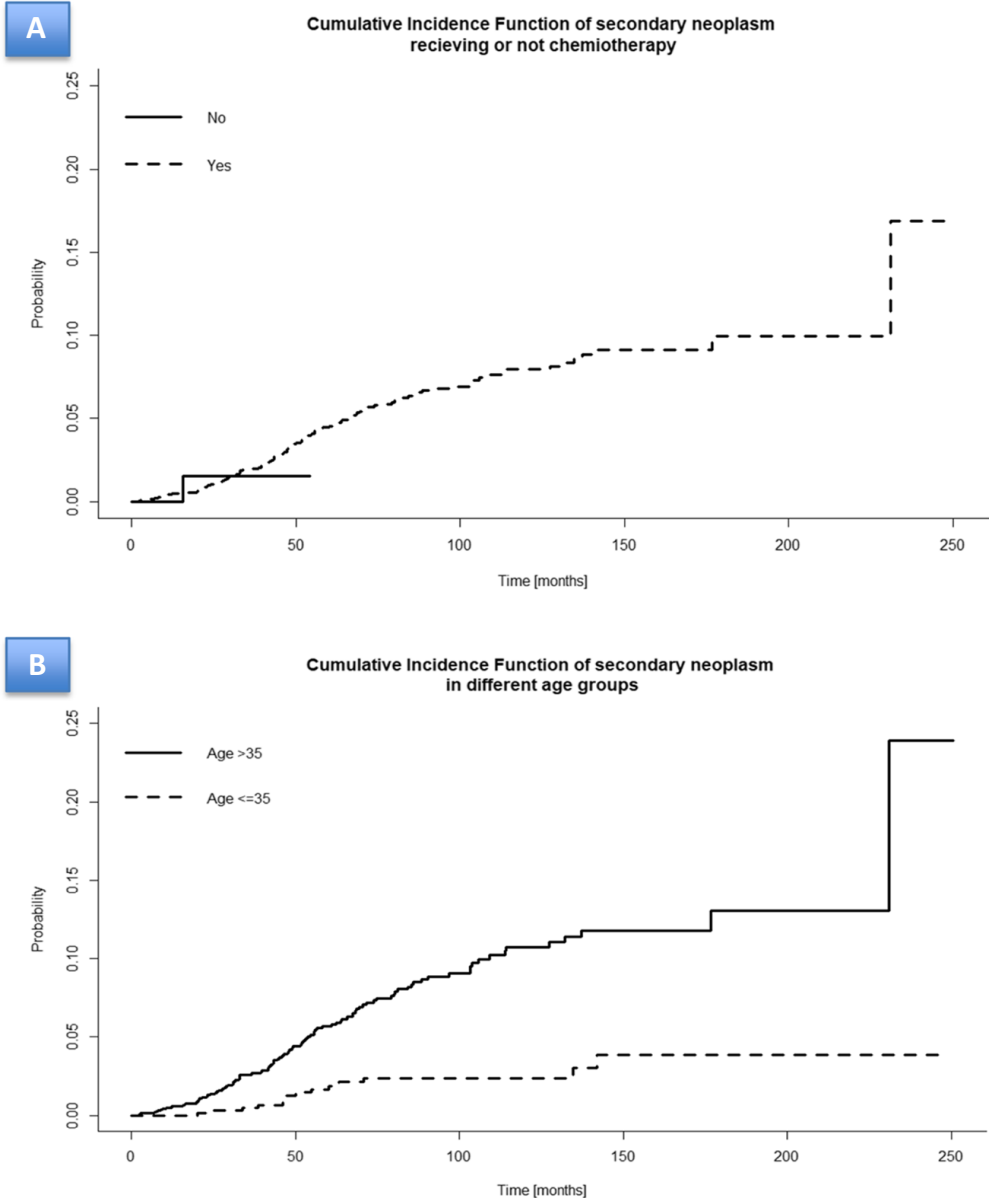
**Cumulative incidence of s-NPLs according to risk factors.** (A) Cumulative incidence of s-NPLs according to type of PETHEMA protocol: chemotherapy-based vs chemotherapy free. (B) Cumulative incidence of s-NPLs according to the age (<35 vs >= 35 y.o.) at diagnosis of APL

The following risk factors were analysed as possible risk factors for development of all s-NPLs and s-MDS/AML and solid tumour separately: age, gender, ECOG and Sanz risk score, fever and coagulopathy, WBC and platelet counts, karyotype t(15;17) vs t(15:17)+others, FLT3-ITD mutation at diagnosis of APL, chemotherapy-based vs chemotherapy free regimens. Multivariate analysis identified only age ≤ 35 years (hazard ratio = 0.2584; *p* < 0.0001) as an independent prognostic factor for all s-NPLs (Table 3, Fig. 3B); we found no significant risk factor for development of s-MDS/AML or solid tumour separately probably due to low number of patients.

Respect to type of therapy, all cases with s-MDS/AML (*n*=117) received chemotherapy-based PETHEMA protocols (APL96 – *n*=14, APL99 – *n*=35, APL2005- *n*=69 and APL2012 – *n*=12). After chemotherapy-free protocol, there was only one case of s-NPL and it was a solid tumour. CI of s-NPLs according to chemotherapy-based vs chemotherapy-free regimens is presented in a Fig. 3A. In multivariate analysis, there were no significant differences of s-NPLs development between chemotherapy-based vs chemotherapy-free regimens (hazard ratio = 1.09; *p* =0.932).

### Outcomes of patients developing s-NPLs

**Fig. 4:**
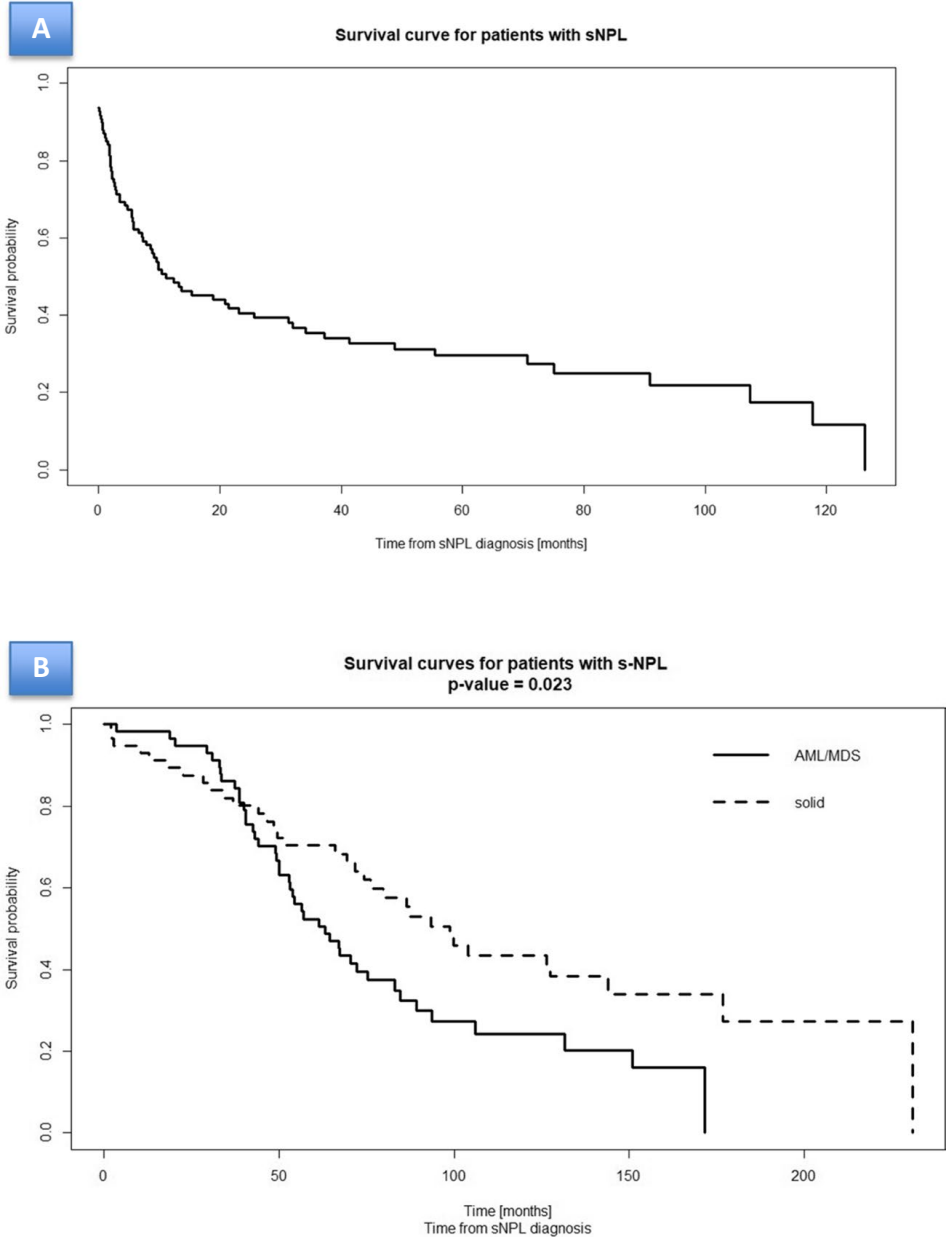
**Overall survival estimated by Kaplan-Meier method according to the occurrence of s-NPLs.** (A) Occurrence of all s-NPLs. (B) Occurrence of s-MDS/AML vs solid tumours

Overall, the 2-year OS from diagnosis of s-NPLs was 40.6%, with a median OS of 11.1 months (Fig. 4A, Table 4). The 2-year OS from diagnosis of s-MDS/AML and from diagnosis of solid tumours was 31.5 % vs 47.4% with median OS of 9.4 months vs 13.7 months respectively (Fig. 4B). In all patients who died, the main cause of death was s-NPLs itself.

**Table 4:** Frequency of deaths and median follow-up of APL patients after development of s-NPLs (according to PETHEMA protocol)

PETHEMA protocol	Death/alive (%)	Median follow-up
APL1996	11/14 (78.6%)	20.6 (6.6 — NA)
APL1999	28/35 (80%)	12.4 (7.4 — 41.4)
APL2005	27/48 (56.3%)	9.1 (3.0 — NA)
APL2012	7/12 (58.3%)	7.9 (3.6 — NA)
APL2017	0/1 (0%)	NA (NA — NA)

## Discussion

Our study shows that s-NPLs, both s-MDS/AML and second solid tumours, represent a relatively frequent and poor prognosis late complication in patients with APL. The only risk factor related with the development of all s-NPLs was older age (> 35 years old at diagnosis of APL). Our data suggest a potential reduction in incidence of s-NPLs after chemotherapy-free regimens. Nevertheless, longer follow-up is needed to affirm that chemotherapy-free protocols could reduce the risk of developing s-NPLs. As APL is currently the most curable subtype of AML, all efforts should be focused on reducing long-term toxicities, including s-NPLs.

Generally, the occurrence of s-NPL in survivors of adult cancer has risen from 9 to 19% of all neoplasms diagnosed in 1975–1979 and in 2005–2009 respectively [39]. In case of APL patients, data respect s-NPL are very rare. According to previous publications, the crude incidence of s-MDS/AML ranges between 0.97 and 6.5% [17, 18], and according to a previous PETHEMA analysis the 6-year CI of s-MDS/AML was 2.2% [14]. This result was confirmed in the present publication (a 5-year CI of s-MDS/AML was of 2.8%), while a 10-year CI was almost double (4.03%). The crude incidence of second solid tumours ranges between 1.52 and 18%, but the number of reports is even more limited [14, 20, 21]. In the present study, with longer follow-up, we enlarge the prior PETHEMA series and we analyse the CI of solid tumours and other hematological malignancies as well, resulting in an overall s-NPLs CI of 4.4% at 5-year CI and 7.9% at 10 years. Of note, we show that with longer follow-up, the CI of s-NPLs will clearly increase, in particular for solid tumours were 10-year CI was more than double that of 5-year CI (3.7% vs 1.5%). Unfortunately, we could not compare our series with an age-matched cohort of non-APL populations, but give the younger median age of APL patients (between 40 and 45 years old), our findings clearly suggest that developing and being treated of APL is leading to a higher risk of s-NPLs.

Like in previous publications [13–23], we corroborate that prognosis of s-NPLs is very poor (40.6% of 2-year OS, with a median OS of 11.1 months) and even worse for s-MDS/AML cases. In case of s-MDS/AML, it is certainly related to the presence of adverse cytogenetic abnormalities observed at time of diagnosis of second neoplasm. Moreover, it could be related to cumulative toxicity of previous therapies, including that for APL. Importantly, in our series, the main cause of death after development of s-NPLs was always related to resistance to therapy, progression of second neoplasm or side effects related with the treatment of s-NPLs.

Risk factors for s-NPLs development are still not clearly defined. In a present study, we observed that s-MDS/AML cases occurred only among APL patients treated with chemotherapy-based regimens. According to previous PETHEMA analyses, the incidence of s-MDS/AML between patients treated with APL96 vs anthracycline-reinforced APL99 protocol was similar. Intermediate risk APL patients obtain higher dose of anthracycline that could be a risk for s-MDS/AML development [14]. Nevertheless, we have not observed correlation between Sanz risk score and all s-NPLs or s-MDS/AML development. The impact of maintenance therapy with methotrexate and mercaptopurine is still not clear [14]; however, all patients (except one treated with APL2017 protocol) who developed s-NPLs, received maintenance therapy. The diagnosis of s-MDS/AML with abnormal karyotype (complex karyotype, abnormalities of chromosome 5 or 7) suggests a close relationship with prior cytotoxic agents [14–18, 24, 25]. Complex karyotype and chromosome 5 and 7 abnormalities are associated with use of alkylating agents. In APL therapy topoisomerase II inhibitors (anthracyclines) are used, which have been shown to be associated with the 11q23 rearrangement (*KMT2A*, previously *MLL*) [24, 25]. In this context, the role of topoisomerase II inhibitors in leukemogenesis of s-MDS/AML in APL patients remains unclear. Moreover, at the time of diagnosis of APL, additional chromosomal abnormalities (trisomy 8 and abnormalities in chromosome 7 among others) are quite frequent (28–30%) [33, 40–42]. Probably, due to low number of patients with additional karyotype, the present study was not able to determine the role of additional chromosomal abnormalities on the development of s-NPL nor s-MDS/AML in APL patients after achieving of CR.

The age more than 35 years old was suggested as an independent risk factor for development of s-MDS/AML [14]. Based on the present analysis, we confirmed that age of 35 or more is an independent risk factor for development for all s-NPLs but not for s-AML/MDS and solid tumours separately.

Data concerning the incidence of s-NPLs after chemotherapy-free regimens are still scarce, but they seem to be equal [20–22] or less frequent than after chemotherapy-based regimens. There is a close correlation between exposure to inorganic arsenic and an increase in lung, kidney, liver, bladder and skin cancers incidence [26, 27]. We were unable to show reduction of s-NPLs after chemotherapy-free regimens compared to AIDA-based protocols probably due to: (1) the limited number of patients treated with chemotherapy-free regimens; and (2) the shorter follow-up of these patients, which is very relevant taking into account that median latency to occurrence of solid tumours overlaps with longer follow-up of ATO+ATRA patients in our series.

The incidental occurrence of s-NPLs in APL patients cannot be ruled out. Many risk factors such as increased body mass index (BMI), diet, cigarette smoking, low physical activity could be responsible for the development of s-NPLs in the general population. Interestingly, the majority (59%) of APL patients are overweight or obese (BMI ≥25) [43].

Our study has several limitations related to retrospective design; there are missing data concerning the molecular and cytogenetical analysis at time of diagnosis of APL and of s-NPLs. The number of patients with s-NPL and in particular the group of patients treated with ATRA plus ATO is very small and with short follow-up period. Despite these limitations, this study was performed on a large group of APL patients treated homogenously.

To conclude, s-NPLs are relatively frequent in APL patients. We report higher incidence than previous reports as patients had substantial follow-up. The development of s-NPLs was associated with dismal outcomes, highlighting the need of preventive strategies, long-term monitoring and high suspicion after APL therapy. Further studies are needed in order to assess the potential reduction of the risk of s-NPLs among patients treated with chemotherapy-free regimens.

### Supplementary information

ESM 1

## Data Availability

The data that support the findings of this study are available from the corresponding author upon reasonable request.
