# Supplementary material for: Incidence, risk factors, and outcomes of second neoplasms in patients with acute promyelocytic leukemia: the PETHEMA-PALG experience
Source: Ann Hematol. 2023 Dec 19;103(2):451–61. doi: 10.1007/s00277-023-05582-y (PMC10799093; doi:10.1007/s00277-023-05582-y)
Supplement: Supplementary file 1 — ESM 1 [file 277_2023_5582_MOESM1_ESM.docx]

**SUPPLEMENTARY FIGURES**


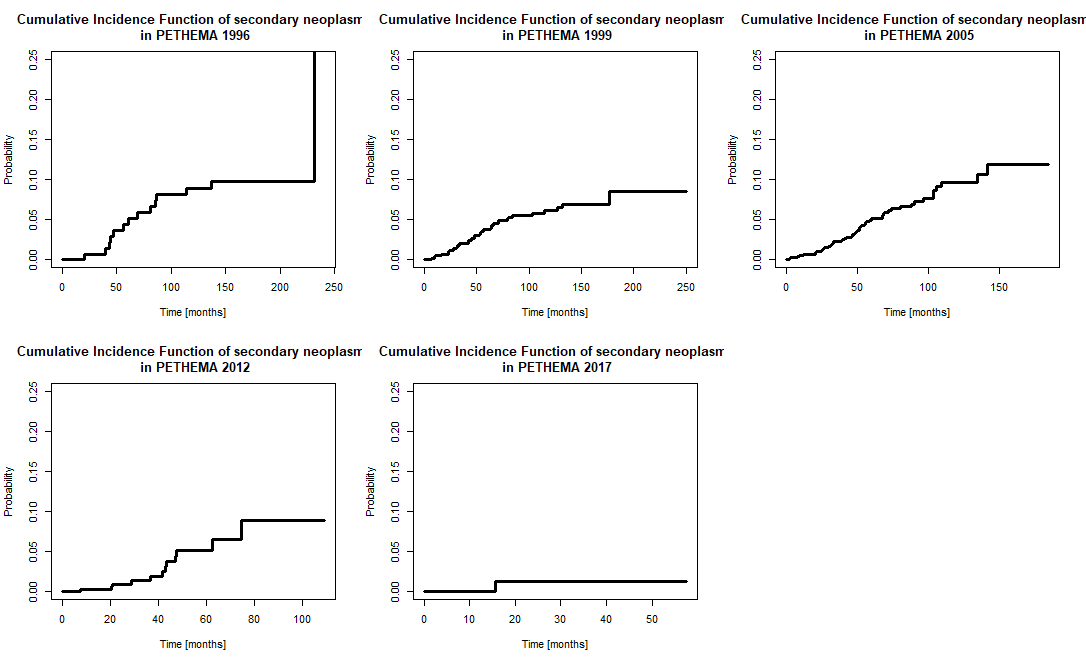
**Supplementary Figure 1.Cumulative incidence according to PETHEMA protocols: 1996, 1999, 2005, 2012 and 2017.**


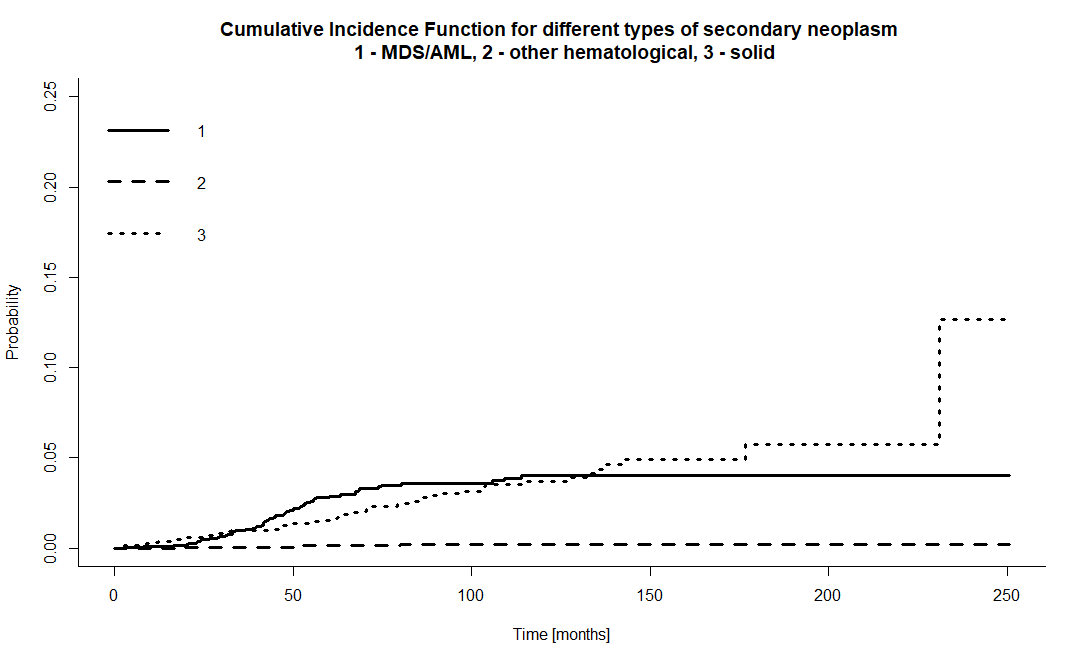


**Supplementary Figure 2. Cumulative incidence of s-NPLs according to type of s-NPLs.**
